# Cap analog and Potato virus A HC-Pro silencing suppressor improve GFP transient expression using an infectious virus vector in *Nicotiana benthamiana*

**Published:** 2017-06

**Authors:** Amin-alah Tahmasebi, Alireza Afsharifar

**Affiliations:** Plant Virology Research Center, Shiraz University, Shiraz, Iran

**Keywords:** Cap analog, HC-Pro, N. *benthamiana*, Recombinant protein

## Abstract

Transient expression of proteins in plants has become a choice to facilitate recombinant protein production with its fast and easy application. On the other hand, host defensive mechanisms have been reported to reduce the efficiency of transient expression in plants. Hence, this study was designed to evaluate the effect of cap analog and Potato virus A helper component proteinase (PVA HC-Pro) on green fluorescent protein (GFP) expression efficiency. *N*.* benthamiana* leaves were inoculated with capped or un-capped RNA transcripts of a Turnip crinkle virus (TCV) construct containing a green fluorescent protein reporter gene (TCV-sGFP) in place of its coat protein (CP) ORF. PVA HC-Pro as a viral suppressor of RNA silencing was infiltrated in trans by *Agrobacterium tumefaciens*, increased the GFP foci diameter to six and even more cells in both capped and un capped treatments. The expression level of GFP in inoculated plants with TCV-sGFP transcript pre-infiltrated with PVA HC-Pro was 12.97-fold higher than the GFP accumulation level in pre-infiltrated leaves with empty plasmid (EP) control. Also, the yield of GFP in inoculated *N. benthamiana* plants with capped TCV-sGFP transcript pre-infiltrated with EP and PVA HC-Pro was 1.54 and 1.2-fold respectively, greater than the level of GFP expressed without cap analog application at 5 days post inoculation (dpi). In addition, the movement of TCV-sGFP was increased in some cells of inoculated leaves with capped transcripts. Results of this study indicated that PVA HC-Pro and mRNA capping can increase GFP expression and its cell to cell movement in *N. benthamiana*.

## INTRODUCTION

There is a significant increase in global demand to produce large amounts of recombinant proteins for medical and biological researches including vaccines, industrial enzymes and components of novel nanoparticles [1, 2]. Plants are one of the most cost effective and safe systems for large-scale production of recombinant proteins. In addition, they are able to modify appropriately post-translation process of expressed recombinant proteins [3]. Transient expression of foreign proteins in plants has been mediated by different assays including viral vector-based transient amplification [4, 3]. Plant viral vectors are easier to manipulate and recombinant proteins can be produced more quickly [5]. The development of a reverse genetics system, and especially the fact that virus genome can be manipulated, has opened new opportunities for studies aimed at exploring the potential of plant viruses as a vector to express foreign proteins. Using of cDNA clones of viruses, like those expressing GFP, greatly facilitates high throughput screening, for generating recombinant proteins [6, 7]. The production of biologically active viral RNA from cDNA clones *in vitro* has become an essential step in the molecular genetic analysis of positive-strand RNA viruses and development of new viral vectors and vaccines [7]. A TCV-sGFP construct containing GFP replaced with CP open reading frame (ORF) was used to express GFP in *N. benthamiana*. TCV is a small icosahedral virus from *Carmovirus* genus, *Tombusviridae* family with a positive-sense RNA genome of 4,054 nt in size and contains four ORFs [8, 9]. Sequences in the 3' terminal untranslated region fold into a Y-shaped structure that control cap-independent translation [9]. Deletion of the CP gene from TCV affects cell-to-cell movement in *N. benthamiana*, hence its incapable of moving cell-to-cell [10-12]. Also, deletion of the TCV movement proteins inhibits movement complementation by TCV CP, indicating that TCV CP does not have an independent movement function [13]. TCV CP has been shown to have a viral suppressor of RNA silencing (VSR) activity [14]. RNA silencing is an important antiviral defense mechanism that directly defends host cells against foreign nucleic acids including viruses [15]. In order to survive in hosts with RNA silencing, viruses have evolved a counter defense by encoding proteins that disrupt the RNA silencing pathway, known as VSR [16]. VSRs interact with the elements of the silencing pathway to block gene silencing [17-19]. Host RNA silencing has been reported to reduce the efficiency of transient expression in plants [20, 21]. Hence, to increase recombinant GFP protein expression, we investigated the effects of exogenous applied 7 methyl guanosine (5') triphospho (5') guanosine (m7GpppG, cap analog) and PVA HC-Pro silencing suppressor. PVA belongs to *Potyviridae* (genus *Potyvirus*) and codes HC-Pro as a multifunctional protein involved in many steps of the viral infection cycle [22, 23]. Additionally, *in vivo*, most mRNA molecules have a cap structure which is critical for efficient translation and protects mRNA from degradation by intracellular nucleases. Also, the cap at mRNA 5' ends provides a specific recognition structure for translation initiation factors to recruit ribosomes to mRNAs during translation initiation [24]. In general, capping process is essential for RNA stability, efficient translation, nuclear transport of mRNA, transcription, polyadenylation and splicing [25-27]. Thus, the main objective of this study was to demonstrate the efficiency of cap analog and PVA HC-Pro silencing suppressor activity on the GFP expression, GFP fluorescent level and cell-to-cell movement of TCV sGFP.

## MATERIALS AND METHODS

**Plant material and growth conditions:** *N. benthamiana *plants were grown with a photoperiod 16 hr light/8 hr dark and minimum temperature of 18°C, and maximum temperature of 22°C and 80% humidity. 

**Plasmid constructs: **The cDNA of HC-Pro ORF of PVA was amplified and cloned into a pDONRTM/Zeo vector (Life technology). Clones were controlled by sequencing and further recombined into the Gateway (Invitrogen) destination vector pGWB17 with C-terminal myc tag and N-terminal 35S promoter for agroinfiltration tests. The Agrobacterium vector pPZP212 containing TCV-sGFP with the *GFP* ORF in place of the *CP* was described before [13]. Also, Agrobacterium strain C58C1 carrying EP was prepared as previously reported [28] and was used as a negative control. Finally, these clones were checked by restriction analysis with *Xba*I and *Sac*I restriction enzymes. Each of these plasmids was introduced into *Agrobacterium tumefaciens* C58C1 by freeze-thaw method. 

**Linearization and purification of pPZP212 containing TCV-sGFP: **Plasmid containing full-length TCV-sGFP was linearized by cleavage with *Xba*׀ enzyme, and incubated 1 hour at 37°C. Then it was run on 1 percent agarose gel. Linearized plasmid DNA was extracted with phenol/chloroform and precipitated in the presence of sodium acetate, and dissolved in RNase-free water. 

***In vitro*** **transcription with T7 RNA polymerase: **Complete *in vitro* transcription reaction mixtures contained 5 to 10 µg of linearized DNA template plus nuclease free water; 25 mM (each) ATP, CTP, and UTP; 2 mM GTP; 40 mM cap analog m7G(5') ppp (5')G (New England Biolabs); 10µl of T7 RNA polymerase; and 20 µl T7 transcription buffer (Promega) in a total volume of 100 µl. *In vitro* transcription by T7 RNA polymerase (Promega) was carried out in the presence or absence of the cap analog under the reaction condition. The reaction mixtures were incubated at 37°C for 2h, and RNA product was analyzed by agarose gel electrophoresis followed by ethidium bromide staining. The concentration of RNA was estimated by UV absorption following ethidium bromide staining. 

**Preparation of Agrobacterium cultures for infiltration: **Agrobacterium cultures harboring PVA HC-Pro and EP constructs were prepared for infiltration. Briefly, individual colonies from PVA HC-Pro and EP constructs were inoculated into 3-ml YEP broth culture in the presence of appropriate antibiotics, kanamycin, carbenicillin, and rifampicin and also, spectinomycin and rifampicin, respectively and were incubated at 28°C for 20 h with shaking. From these initial cultures, 1 ml was used to inoculate 50-ml YEP broth cultures with the antibiotics supplemented with acetosyringone (20 µM final concentration) and 0.5 M MES, pH 5.6. These cultures were similarly incubated at 28°C for 20 h with shaking. Upon reaching OD_600_ ;0.5, cultures were subsequently pelleted by centrifugation at 3500g for 5 min and re-suspended in induction buffer containing 10 mM MgCl_2_, 10 mM MES, pH 5.6, and 150 µM acetosyringone and incubated for at least 2 h prior at room temperature with constant rotation. Agrobacterium cultures with OD_600_; 0.5 diluted in induction buffer containing the PVA-HC-Pro or EP constructs (final OD_600_= 0.5) were syringe-infiltrated into the abaxial side of *N. benthamiana* leaves at six- to eight-week-stage. 

**Inoculation of plants: **Wild type *N. benthamiana* plants were used for agroinfiltration tests as described previously [29]. After 24 h post infiltration with *A. tumefaciens* strains carrying PVA-HC-Pro or EP constructs, the abaxial surfaces of fully expanded leaves of the 6-week-old *N. benthamiana* plants were mechanically inoculated with TCV-sGFP RNA transcripts diluted in 3X GKP buffer (50 mM glycine; 30 mM K_2_HPO_4_, pH 9.2; 1% bentonite; 1% celite) [30]. Six plants were inoculated with transcripts containing cap or without cap analog in GKP buffer for each experiment. Immediately after inoculation, plants were rinsed with water and placed in a growth chamber under light conditions (photoperiod 16 hr light/8 hr dark and minimum temperature of 18°C, and maximum temperature of 22°C) and approximately 80% humidity. Detection of GFP fluorescence in detached *N. benthamiana* leaves was performed using a hand-held long-wave UV lamp UVL-56 (UV Products). Each assay was repeated three times. The number of infected plants was determined via observing GFP fluorescent using UV lamp after inoculation at 2 dpi and expressed as an infectivity percent of total inoculated plants with TCV-sGFP in the presence or without cap analog.

The infection process was evaluated at 5 dpi, with a Leica MZIII fluorescence dissecting microscope equipped with a GFP filter. The movement of TCV-sGFP and its foci diameter was measured [13]. Sample preparation and light microscopy were performed as described previously [28]. Fluorescence microscopy was conducted as described [31]. 

**RNA extraction, cDNA Synthesis and qPCR: **Total RNAs were extracted from infiltrated leaves with the spectrum plant total RNA kit (Sigma-Aldrich) and on-column DNaseI Digest Set (Sigma-Aldrich) according to the manufacturer’s instructions. Total RNA concentrations were measured with a ND-1000 spectrophotometer (Nanodrop, Wilmington, DE, U.S.A.), and one-microgram aliquots of total RNA samples were used for cDNA synthesis with iScript reverse transcriptase first-strand cDNA synthesis kit (MBI Fermentas). Primers amplifying the *EF-1α* and *GFP* were used as control and target for equal cDNA amounts (Table 1). The cDNA was quantified with the DyNAmo Flash SYBR Green qPCR kit (Finnzymes) using a Bio-Rad IQ5 icycler apparatus according to the manufacturer’s recommendations. PCR was run in 96-well optical reaction plates (Bio-Rad). The PCR conditions were as follows: 95°C for 7 min to activate hot start Taq DNA polymerase, followed by 40 cycles of 95° C for 10 s and 60°C for 30 s. At the end of the PCR, dissociation kinetics analysis was performed to check the specificity of annealing. Three biological replicates were used, and three technical replicates were performed for each biological replicate. Accordingly, the fold expression of target mRNAs over the reference values were calculated by the equation 2^-DDCT^ [32], where DCT was determined by subtracting the corresponding internal control CT value from the specific CT of the target (GFP), and DDCT was obtained by subtracting the DCT of each experimental sample from that of the control sample.

**Table 1:** Details of specific primers used in this study

**Primer**	**Sequence from 5′ to 3′ **	**Product size (bp)**
EF-1α-F*	AGCTTTACCTCCCAAGTCATC	116
EF-1α-R*	AGAACGCCTGTCAATCTTGG	
GFP-F	TCCATGGCCAACACTTGTCA	102
GFP-R	GGCATGGCACTCTTGAAAAAG	

* F: Forward strand, R: Reverse strand

**Western blot Analysis for GFP and HC-Pro proteins: **Leaf samples (100 mg fresh weight) from infiltrated and inoculated *N. benthamiana* leaves at 4 days post infiltration were ground and homogenized in 300 µl of protein laemmli buffer (0.125 M Tris–HCl, pH, 6.8; 4% SDS; 10% 2 mercaptoethanol; 20% glycerol; 0.004% bromophenol blue), heated at 95°C for 5 min, and centrifuged (10 min at 13,000 rpm) to remove insoluble material. The supernatant was run on a 12% SDS-PAGE and after electrophoresis, proteins were transferred onto a PVDF membrane as described [33] and blocked with 5% (w/v) nonfat milk powder in phosphate-buffered saline containing 0.05% (v/v) Tween 20. Detection was carried out with primary mouse monoclonal antibody (dilution 1:1,000 in PBS-T plus 2.5% milk) and secondary anti mouse polyclonal antibody (dilution 1:5000 in PBS-T plus 2.5% milk) and signals detected using the ECL Prime kit (Amersham, GE Healthcare) with the enhanced Chemiluminescence plus protein detection system (Amersham). They were imaged in a LAS-3000 Luminescent Image Analyzer (Fujifilm, Fuji Photo Film, Kleve, Germany). HC-Pro and GFP proteins were detected using myc-specific monoclonal antibodies and GFP-specific antiserum (Sigma), respectively.

**Statistical analysis: **The experimental data were statistically analyzed using analysis of variance (ANOVA) with subsequent Duncan’s multiple range test [34] using SAS 9.4 software. The differences at the 95% confidence level were considered to be significant.

## RESULTS

**Figure 1:**
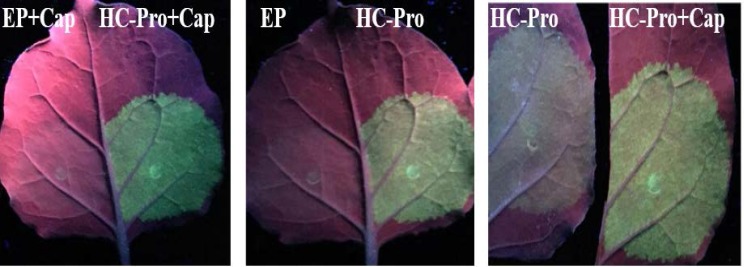
Leaves of *N. benthamiana* which divided into two equal parts, infiltrated with *A. tumefaciens* cells harboring pGWB17 PVA HC-Pro and opposite side of leaf infiltrated with pGWB EP, then inoculated with TCV-sGFP transcript in the presence or without cap analog. Detection of GFP fluorescence in detached *N. benthamiana* leaves was performed using a hand-held long-wave UV lamp UVL-56 (UV Products) at 5 dpi

To evaluate the effect of cap analog and PVA HC-Pro silencing suppressor on expression level of *GFP* gene, an infectious TCV-sGFP transcript containing GFP replaced with CP ORF was used. Pre-infiltrated *N. benthamiana* leaves with HC-Pro or EP were inoculated with the capped or un-capped RNA transcript of TCV-sGFP. HC-Pro and cap analog application showed stronger GFP green color under UV lamp in leaves (Fig 1).

**Figure 2:**
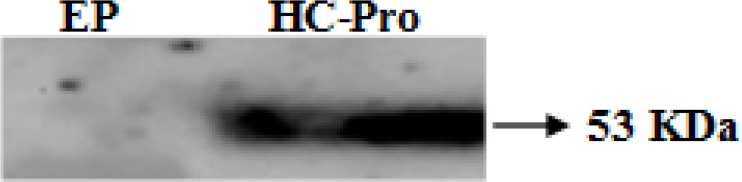
Western blot analysis of extracts from infiltrated *N. benthamiana* plants with EP and PVA HC-Pro at 5 dpi. Signal was detected using the ECL Prime kit (Amersham, GE Healthcare) with the enhanced Chemiluminescence plus protein detection system (Amersham). It was imaged in a LAS-3000 Luminescent Image Analyzer (Fujifilm, Fuji Photo Film, Kleve, Germany). HC-Pro protein was detected using myc-specific monoclonal antibody (Sigma). Also, EP construct was used as a negative control

In western blot analysis, HC-Pro protein, with an apparent molecular weight of 53 kDa, was detected in infiltrated leaves with agrobacterium culture harboring PVA HC-Pro. In contrast, no protein was detected in extracts prepared from plants infiltrated with agrobacterium carrying an EP (Fig. 2).

**Figure 3:**
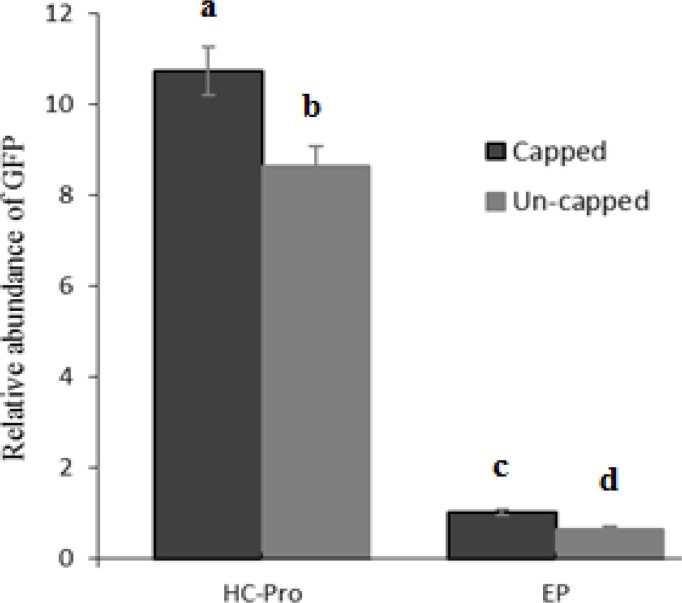
GFP accumulation level in infiltrated leaves with a PVA HC-Pro and EP constructs, then inoculated by TCV-sGFP in the presence or absence of cap analog was measured by qRT-PCR. Mean and SD were obtained from three biological replicates. Vertical bars represent standard deviation (SD). Means are statistically significant at p<0.05, when they share no common letter

The expression level of GFP in *N. benthamiana* plants inoculated with TCV-sGFP transcript pre-infiltrated with PVA HC-Pro was 12.97-fold greater than the level of GFP mRNA expression achieved in the same leaves pre-infiltrated with EP. The suppressor activity of PVA HC-Pro delayed the plant-derived silencing effect which can be monitored by enhancement of transiently expressed GFP marker. In this experiment, GFP expression was estimated to be 1.54 and 1.2-fold higher in the presence of cap analog pre-infiltrated with EP and PVA HC-Pro than the value of GFP transcript without cap analog at 5 dpi (Fig. 3).

**Figure 4:**
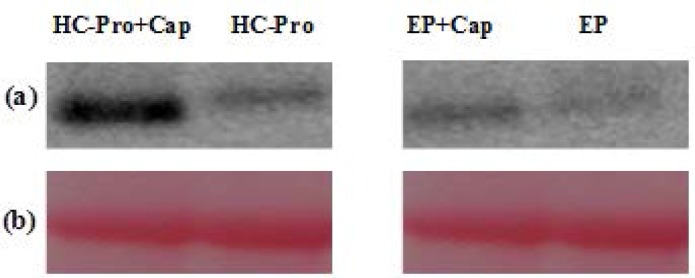
Western blot analysis of GFP protein. (a) Samples were extracted from leaf tissues agroinfiltrated with PVA HC-Pro and subsequently inoculated with TCV-sGFP in the presence of cap analog or inoculated with TCV-sGFP without cap analog. Extracts from infiltrated leaves with EP were used as control. Then, they were inoculated with TCV-sGFP in the presence of cap analog or inoculated with TCV-sGFP without cap analog. A total of 30 μg extracts for each sample were prepared at 5 dpi and subjected to western blot analysis with anti-GFP antibodies. (b) Ponceau S staining of the large subunit of Rubisco was used as a loading control (bottom panel

To compare GFP expression levels from TCV-sGFP transcript in the presence of cap analog pre-infiltrated with EP and PVA HC-Pro, western blot analysis was performed using extracts prepared from inoculated leaves. This analysis confirmed the enhancement of GFP RNA expression in the presence of PVA HC-Pro and cap analog (Fig. 4). 

**Figure 5:**
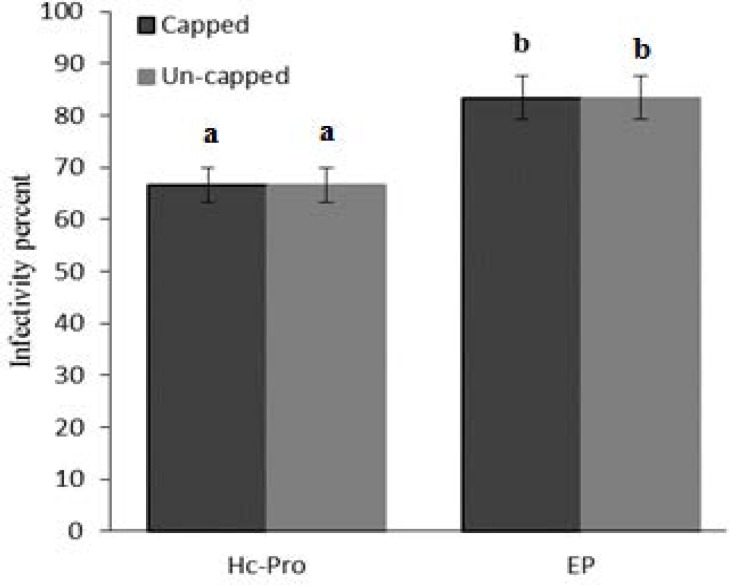
The effect of cap analog on infectivity rate of TCV-sGFP in leaves pre-infiltrated with PVA HC-Pro or EP. The infectivity rate was determined from six biological replicates using UV lamp at 2 dpi. Vertical bars represent SD. Means are statistically significant at p < 0.05, when they share no common letter. The comparisons were made using the Duncan test

**Figure 6:**
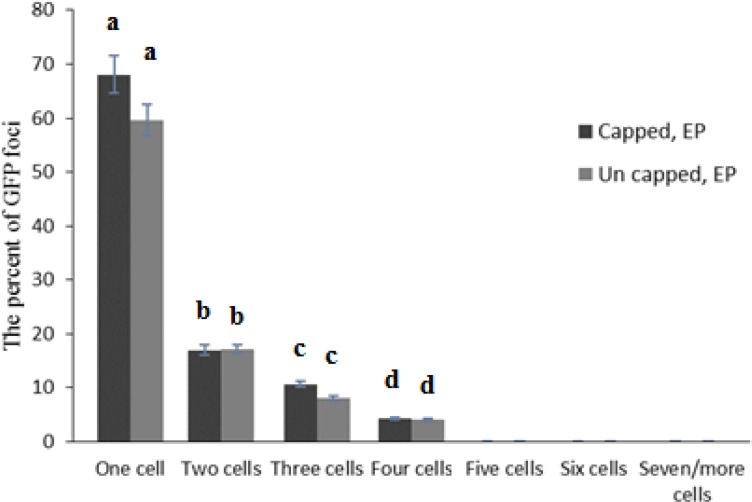
Agroinfiltration of EP, and subsequent inoculation with TCV-sGFP in the presence or absence of cap analog. Total number of fluorescent foci counted. Number of foci in which TCV-sGFP was confined to a single cell, or was found in two adjacent cells, in a cluster of three cells or in a cluster of four cells. Vertical bars represent SD. Means are statistically significant at p < 0.05, when they share no common letter. The comparisons were made using the Duncan test

**Figure 7:**
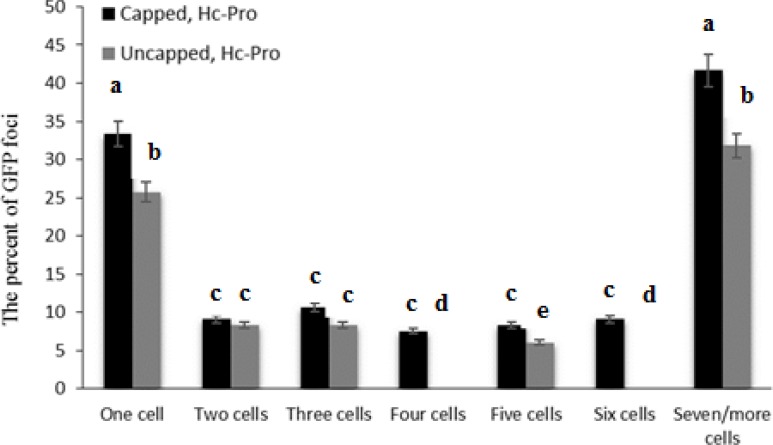
Agroinfiltration of HC-Pro, and subsequent inoculation with TCV-sGFP in the presence or absence of cap analog established the validity of the leaf-based VSR complementation assay. GFP expressed from the TCV genome. Total number of fluorescent foci counted at 5 dpi for constructs. Number of foci in which TCV-sGFP was confined to a single cell, or was found in two adjacent cells, in a cluster of three cells or in a cluster of four or more cells. Vertical bars represent SD. Means are statistically significant at p < 0.05, when they share no common letter. The comparisons were made using the Duncan test

Infectivity rate of pre-infiltrated leaves with HC-Pro and EP in the presence or without cap analog was 66.7 and 83.3 percent, respectively (Fig. 5). Cap analog could increase TCV-sGFP movement in some cells infiltrated with PVA HC-Pro at 5 dpi. Analysis of cell movement in either capped RNA transcript or uncapped transcript of TCV-sGFP showed that compared with PVA-HC-Pro, in leaves infiltrated with EP, TCV-sGFP movement was greatly reduced, but one- to four-cell foci of infection was regularly observed (Figs 6,7). Delivery of PVA HC-Pro in trans consistently enhanced GFP expression and complemented movement of the TCV-sGFP to six and even more cells in both capped and uncapped reactions (Fig. 7). 

**Figure 8:**
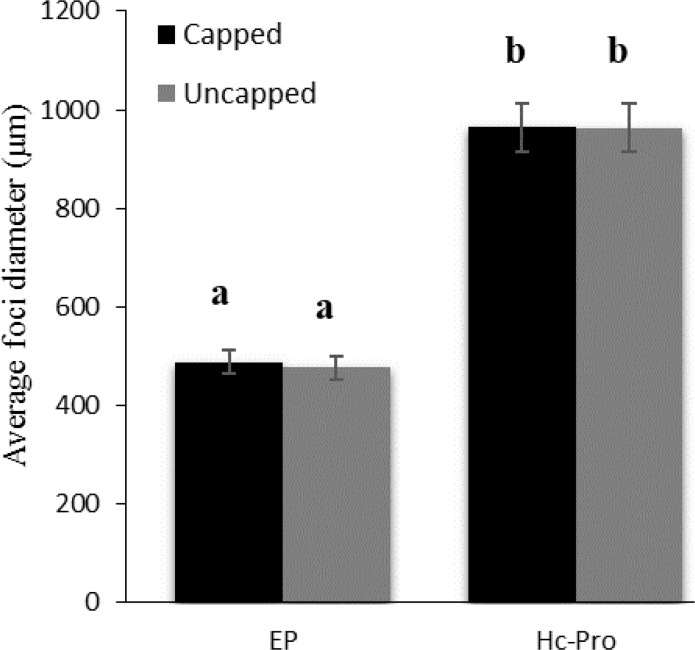
Quantification of the cell-to-cell movement of TCV sGFP in the complementation assay through measurement of the diameter of foci of infection at 5 dpi for PVA HC-Pro and EP constructs infiltrated in the presence or without cap analog. Vertical bars represent SD. Means are statistically different at p<0.05, when they share no common letter. The comparisons were made using the Duncan tes

**Figure 9:**
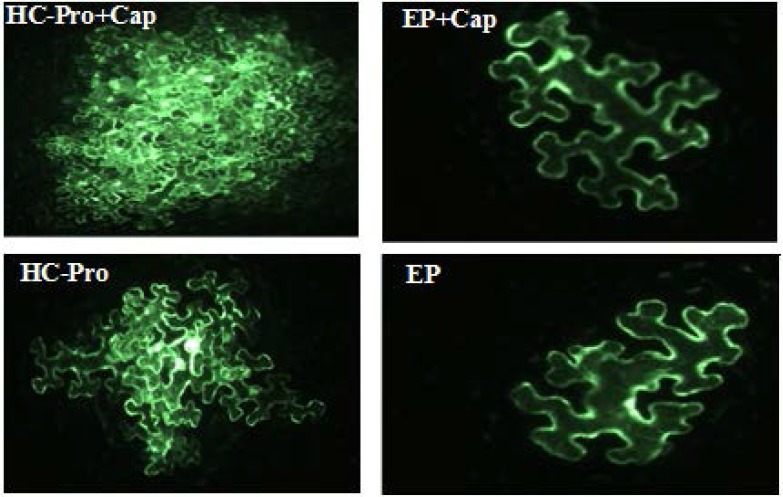
Green-color imaging of the TCV-sGFP complementation assay with constructs expressing PVA HC-Pro or EP. Pre-infiltrated Agrobacterium cultures carrying an EP and subsequently inoculated with TCV-sGFP in the presence of cap analog or inoculated with TCV-sGFP without cap analog did not complement the movement of TCV-sGFP out of the single cell. While, pre-infiltrated PVA HC-Pro construct and subsequently inoculated with TCV-sGFP in the presence of cap analog or inoculated with TCV-sGFP without cap analog increased the movement of TCV-sGFP to neighboring cells. Compared to PVA HC-Pro activity, EP construct was used as a negative control

The average size of GFP foci showed that cap analog cannot affect the average diameter of GFP foci in the presence of PVA HC-Pro. Interestingly, the average size of GFP foci with and without cap analog on pre-infiltrated leaves with the EP was 487 and 477 µm in diameter, while addition of PVA-HC-Pro in trans, significantly increased the diameter of the infection foci (over 964 and 963 µm, respectively) (Figs 8, 9). 

## DISCUSSION

The similar infectivity rate of capped and un-capped transcript was in accordance with another research which showed that the cap structure is not required for infectivity of viral RNA of members of the family *Tombusviridae* [35]. While, the phenomenon of reduced infectivity of uncapped transcripts has been shown for several viruses such as tobacco mosaic virus strain L [36], white clover mosaic virus [37], cymbidium ringspot virus [38] cucumber mosaic virus [39]. The effect of cap analog on GFP protein level can be due to its interaction with eukaryotic translation initiation factor eIF4F to increase GFP protein translation [24]. Also, enhancement of GFP transcript level by capping process might be as a result of increasing in mRNA stability by protecting transcripts from degradation [40, 41]. In general, there was a correlation between the qPCR analysis of GFP accumulation with GFP primers (Table 1) and the photon emission resulted in the respective agroinfiltrated patches by fluorescent microscope. It seems that, the effect of HC-Pro on enhancement of GFP level can be because of its strong RNA silencing suppressor activity to avoid host RNA silencing mechanism. Similar results were obtained previously when GFP was transiently expressed, and the enhancement was ascribed to the stabilization of GFP mRNA by suppression of RNA silencing. The p19 suppressor protein of tomato bushy stunt virus (TBSV) enhanced GFP expression 50 times higher than the level from a control [21]. In another experiment using beet curly top virus replicon-based vector system for transient expression of GFP, in the presence of p19 protein, GFP expression was increased up to 240% (3-fold more than that from control). Additionally, Western blot analysis of this study indicated that GFP levels in the presence of tobacco etch virus HC-Pro and TBSV p19 are 1.1 and 2.4-fold higher, respectively than the levels in a control with no suppressor [42]. Specific alterations and gene replacement experiments in viral genome will allow the study of the replication, symptom development, host range and cis acting elements required for replication and assembly. Taken together, cap analog and PVA HC-Pro were shown to be useful to enhance and extend GFP-expression in *N. benthamiana*, making them potent candidates in small and large scale plant expression systems to allow high foreign protein yield. 
